# Beyond the Bench: ToxRAP Makes the Connection

**DOI:** 10.1289/ehp.113-a236a

**Published:** 2005-04

**Authors:** Tanya Tillett

Environmental health professionals are made, not born. That’s the ideology behind the creation of ToxRAP™ (Toxicology, Risk Assessment, and Pollution), an interactive curriculum for grades kindergarten through 9. ToxRAP was designed to help students understand how elements of the environment around them affect their health. The lessons were developed by the Community Outreach and Education Program (COEP) of the NIEHS Center of Excellence located at the Environmental and Occupational Health Sciences Institute, jointly sponsored by Rutgers and the University of Medicine and Dentistry of New Jersey–Robert Wood Johnson Medical School.

The core of the curriculum design is the ToxRAP Framework, which structures each lesson in a way that enables students to learn concepts central to the areas of toxicology, environmental health, risk assessment, and risk management. Under the framework, students identify a health problem, investigate the problem, reach a conclusion about what’s causing the problem, then figure out how to control the hazard. For example, in the lesson The Case of the WTC Dust, middle school students investigate health risks faced by fictitious New York City residents in ridding their building of toxic dust after the 2001 collapse of the World Trade Center.

When integrating the ToxRAP curriculum into their lesson plans, teachers use three modules that can be taught sequentially or independently of each other. They use math, health, science, and language arts activities that encourage the collection and organization of real information to be evaluated and analyzed using scientific evidence. Teaching tools and techniques include illustrated stories, games, graphing, cooperative group exercises, hands-on experiments, and case studies.

Currently, ToxRAP reaches approximately 4,300 classrooms in 23 states, and the developers have recently trained teachers in Guam and Puerto Rico as well. In addition to the English modules, instructors in bilingual or English as a Second Language classrooms can also receive select portions of course content in Spanish.

According to Laura Hemminger, codirector of the COEP, what makes the program unique is that it teaches younger students to independently evaluate environmental health problems, which empowers them to take some control of their own environmental exposures. Teachers admire the curriculum’s ability to facilitate the building of students’ critical thinking skills by making the learning experience challenging and entertaining.

“ToxRAP is an excellent instructional model. It teaches children every aspect of the scientific method in an enjoyable, interesting way. When I tell students that it is time for science, they applaud! They take out their detective badges and notebooks, and they are ready to go to work,” says Laura Steinberg, a fifth-grade teacher in Fords, New Jersey.

The developers of ToxRAP continue to expand the program, and are currently collaborating with an NIEHS small business grantee to create interactive activities to enhance ToxRAP’s utility in the classroom, and to make it available to students and their families at home. Also on the horizon is a website, additional curricular modules, and the use of student evaluations on the program’s materials to shape future curriculum content.

## Figures and Tables

**Figure:**
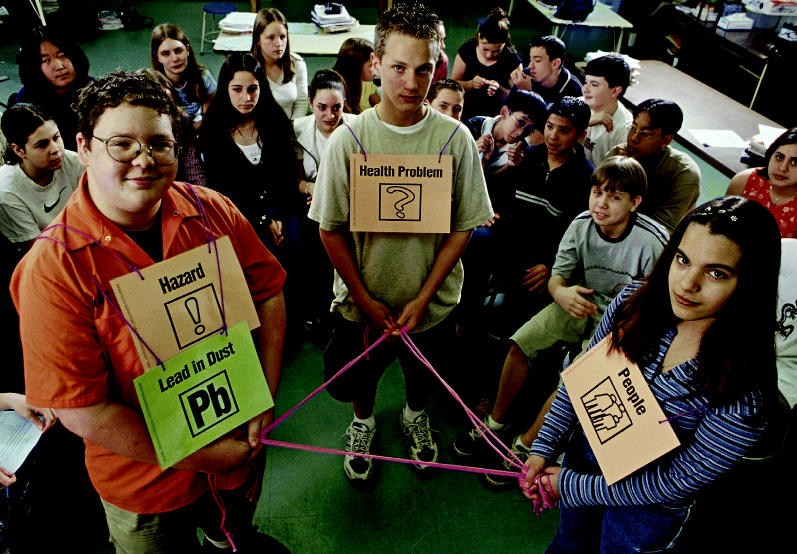

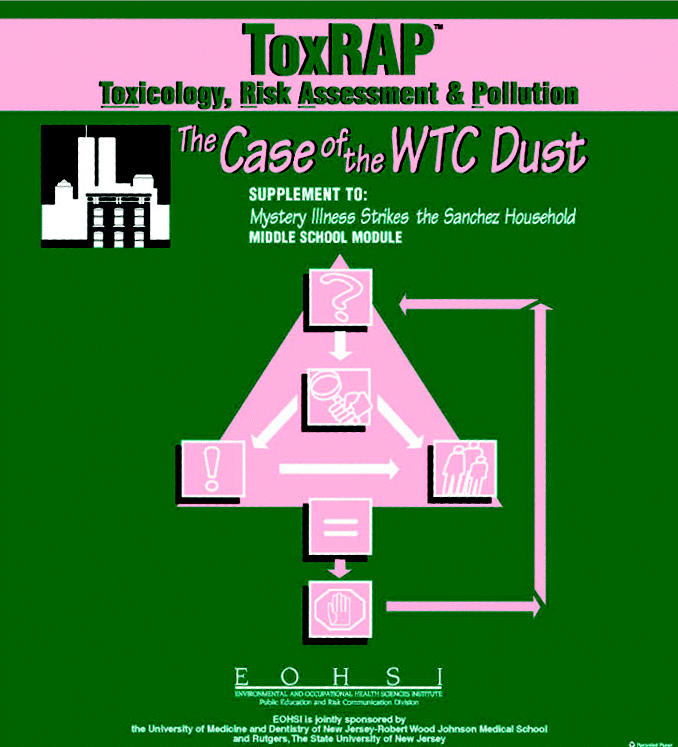
**Learning for life.** The ToxRAP curriculum teaches young students about the interconnections between enviromental exposures and human health.

